# *Mycobacterium senegalense* Infection in Kidney Transplant Patient with Diabetes, Memphis, Tennessee, USA

**DOI:** 10.3201/eid3001.231013

**Published:** 2024-01

**Authors:** Nupur Singh, Reeti Khare, Shirin Mazumder

**Affiliations:** University of Tennessee Health Science Center, Memphis, Tennessee, USA (N. Singh, S. Mazumder);; National Jewish Health, Denver, Colorado, USA (R. Khare);; Methodist University Hospital, Memphis (S. Mazumder)

**Keywords:** bacteria, mycobacterial infections, infectious disease, *Mycobacterium senegalense*, *Mycobacterium conceptionense*, Tennessee, United States, tuberculosis and other mycobacteria

## Abstract

Fewer than 30 cases of *Mycobacterium senegalense* infection have been reported. We report a complicated case of *M. senegalense* infection in Memphis, Tennessee, in the southeastern United States. The patient's comorbidities of past organ transplant and insulin-dependent diabetes required delicate consideration of those health conditions to guide treatment.

*Mycobacterium senegalense*, also referred to as *M. conceptionense*, is a nonpigmented rapid-growing mycobacterium belonging to the *M. fortuitum* group, which was first isolated in 2006 from a posttraumatic osteitis inflammation in France (*1**,**2*). Only a handful of *M. conceptionense* cases are readily identifiable in existing literature. Infections can manifest with pulmonary involvement but more commonly manifest as skin or subcutaneous infection, such as after face rejuvenation surgery, breast augmentation surgery, gastric carcinoma resection, or subcutaneous ankle infection (*3*–*6*). Mycobacterial species like *M. senegalense* have been found in irrigation systems, soil, domestic and wild animals, and dairy products (*7**,**8*). Cases of *M. senegalense* infection have been observed in France, Iran, Taiwan, South Korea, Japan, and the United States, demonstrating an unidentifiable pattern of regional bacterial prevalence.

Establishing an accurate diagnosis of *M. senegalense* infection is incredibly difficult, requiring histological examination and extensive mycobacterial cultures (*9*). The limited susceptibility data also mean an optimal therapy has not been completely established, which leaves certain patient populations, particularly the elderly and immunocompromised, susceptible to increased illness and death from *M. senegalense* infection (*10*). We report a complicated case of *M. senegalense* infection in a patient with a previous kidney transplant and insulin-dependent diabetes mellitus in Memphis, Tennessee, USA.

A 70-year-old Black woman with end-stage kidney disease sought care for a painful, swollen, abdominal wall abscess. She had first noticed the lesion ≈3 weeks before in the left mid-abdomen, where she frequently injected insulin. The patient denied any recent travel, drainage at the site, or fever. The 7- × 4-cm abscess was drained the next day without complications, and we sent the custard-like purulent material for laboratory testing.

The patient had undergone a right-sided cadaveric renal transplant 8 years before; her immunosuppressive regimen consisted of tacrolimus, mycophenolate, and prednisone. The patient’s past diagnoses at the time of infection included type 2 diabetes mellitus, lupus, hypertension, hyperlipidemia, sleep apnea, and coronary artery disease. Insulin injections create small open wounds where pathogens can enter and cause infection. An environmental source of the infection was not sought. The hospital microbiology laboratory detected acid-fast bacilli on direct AFB smear. The patient immediately began empiric antimicrobial drugs, including doxycycline (100 mg 2×/d) and levofloxacin (250 mg 1×/d), adjusted for her creatinine clearance.

The isolate was sent to the National Jewish Mycobacteriology Reference Laboratory (Denver, Colorado, USA) for confirmation and susceptibility testing. Sanger sequencing analysis was performed; BLAST (https://blast.ncbi.nlm.nih.gov/Blast.cgi) testing of the *rpoB* sequence results against the public GenBank database identified *M. senegalense* (>99% homology to existing sequences). A line probe assay for common nontuberculous mycobacteria, GenoType NTM-DR (Hain Lifescience, https://www.hain-lifescience.de), was performed first to differentiate qualitatively and in vitro species of several strains of mycobacteria, such as *M. avium* complex, *M. abscessus*, *M. chelonae*, *M. intracellulare*, *M. chimaera*, *M. massiliense*, *M. bolletii*, and *M. chelonae*, but did not yield a species-level identification. Those tests supported identification as *M. senegalense,* and the sequence was deposited in GenBank (accession no. OR644277). In vitro susceptibility testing demonstrated the antibiotics to which the isolate was susceptible, intermediate, and resistant, according to Clinical and Laboratory Standards Institute guidelines (https://clsi.org) (Table).

**Table:** Antibiotic susceptibility testing for *Mycobacterium conceptionense* sample from kidney transplant patient with diabetes, Memphis, Tennessee, USA*

Antibiotic	MIC, µg/mL	Interpretation
Amikacin IV	<8	S
Amoxicillin/clavulanate	8/4	NI
Azithromycin	<16	NI
Cefepime	>32	NI
Cefotaxime	>64	NI
Cefoxitin	<16	S
Ceftriaxone	>64	NI
Ciprofloxacin	<1	S
Clarithromycin	<0.25	S
Clofazimine	<0.5	NI
Clofazimine/amikacin†	<0.5/2	NI
Doxycycline	<1	S
Gentamicin	<2	NI
Imipenem	<2	S
Kanamycin	<8	NI
Linezolid	4	S
Minocycline	<1	NI
Moxifloxacin	<0.5	S
Tigecycline	<0.25	NI
Tobramycin	4	I
Trimethoprim/sulfamethoxazole	1/19	S

*I, intermediate; NI, no Clinical Laboratory Standards Institute guidelines for this antibiotic/organism combination; S, susceptible.  †Clofazimine is not available as a combined medication commercially in the United States. It is available in the National Jewish Mycobacteriology Reference Laboratory for testing purposes only.

The patient continued taking doxycycline. Levofloxacin was stopped after the patient reported nausea and vomiting. Because of increasing creatinine levels, trimethoprim/sulfamethoxazole was not used. The isolate was susceptible to clarithromycin, but it was not selected because of ongoing tacrolimus treatment. Amoxicillin/clavulanate was chosen, despite the isolate’s intermediate susceptibility, because of better patient tolerance. The final drug regimen was doxycycline (100 mg 2×/d) and amoxicillin/clavulanate (250/125 mg 2×/d) adjusted for renal function; expected treatment course was 4–6 months. At the 3-month clinic follow-up, the lesion had notably shrunk to a 3- × 0.8-cm open wound with no drainage, foul odor, or tenderness. By 6-month follow-up, the lesion had closed and was hyperpigmented and flat, without fluctuance or signs of active infection.

We achieved identification of *M. senegalense* through *rpoB* gene sequencing, and treatment was guided by antibiotic susceptibility options. Separating *M. senegalense* from the rest of the *M. fortuitum* complex species helps guide appropriate treatment and epidemiologic analysis of mycobacterial species by geographic location. This patient presented a unique and delicate case in which adequate treatment required thorough consideration of other medications, diagnoses, and comorbidities. Overall, this complicated, interesting case of *M. senegalense* infection at the site of insulin injections for a diabetic patient in the southeastern United States adds to the limited body of *M. senegalense* infection and treatment knowledge. This case highlights that *M. senegalense* is present in this region, suggesting a higher index of suspicion is needed for patients in those areas. 
